# Stress-induced expression of *IPT* gene in transgenic wheat reduces grain yield penalty under drought

**DOI:** 10.1186/s43141-021-00171-w

**Published:** 2021-05-10

**Authors:** Ailin Beznec, Paula Faccio, Daniel J. Miralles, Leonor G. Abeledo, Cecilia Decima Oneto, María de Belén Garibotto, Germán Gonzalez, Federico Moreyra, Matías Elizondo, Mónica Ruíz, Dalia Lewi, Eduardo Blumwald, Berta Llorente, Antonio Díaz Paleo, Ezequiel Bossio

**Affiliations:** 1 Instituto de Genética, “Edwald A. Favret”, Instituto Nacional de Tecnología Agropecuaria (INTA), Buenos Aires, Argentina; 2 Cátedra de Cerealicultura, Facultad de Agronomía de la Universidad de Buenos Aires, Av. San Martín 4453, Buenos Aires, Argentina; 3 IFEVA, CABA, Buenos Aires, Argentina; 4 Laboratorio de Agrobiotecnología, EEA Balcarce, INTA, Ruta 226, Km 73.5, B7620 Balcarce, Argentina; 5 Consejo Nacional de Investigaciones Científicas y Técnicas (CONICET), C1425FQB, Godoy Cruz 2290, CABA, Buenos Aires, Argentina; 6 EEA Bordenave, INTA, Buenos Aires, Argentina; 7 EEA San Juan, INTA, San Juan, Argentina; 8 Unidad Integrada INTA-UNSJ Dpto. Ing., San Juan, Argentina; 9 Department of Plant Sciences, University of California, Davis, USA; 10 Departamento de Ciencias Básicas, Universidad Nacional de Luján, Buenos Aires, Argentina; 11 EEA Pergamino, INTA, Buenos Aires, Argentina

**Keywords:** Agronomic traits, Cytokinin, Genetic transformation, Isopentenyl transferase gene, Water-deficit tolerance, *Triticum aestivum* L

## Abstract

**Background:**

The heterologous expression of isopentenyl transferase (IPT) under the transcriptional control of the senescence-associated receptor-like kinase (SARK) promoter delayed cellular senescence and, through it, increased drought tolerance in plants. To evaluate the effect of pSARK::IPT expression in bread wheat, six independent transgenic events were obtained through the biolistic method and evaluated transgene expression, phenology, grain yield and physiological biomass components in plants grown under both drought and well-irrigating conditions. Experiments were performed at different levels: (i) pots and (ii) microplots inside a biosafety greenhouse, as well as under (iii) field conditions.

**Results:**

Two transgenic events, called TR1 and TR4, outperformed the wild-type control under drought conditions. Transgenic plants showed higher yield under both greenhouse and field conditions, which was positively correlated to grain number (given by more spikes and grains per spike) than wild type. Interestingly, this yield advantage of the transgenic events was observed under both drought and well-watered conditions.

**Conclusions:**

The results obtained allow us to conclude that the SARK promoter-regulated expression of the IPT gene in bread wheat not only reduced the yield penalty produced by water stress but also led to improved productivity under well-watered conditions.

**Supplementary Information:**

The online version contains supplementary material available at 10.1186/s43141-021-00171-w.

## Background

In cereal crops, abiotic stresses as water deficit, high temperatures, and salinity are the main causes of yield losses. In the coming decades, the frequency of these stresses will increase due to the effects of global climate change [1, 2]. In wheat, as in many other cereals, water-deficit stress is devastating for grain production, with significant negative economic and sociological impacts [3–5]. From 1980 to 2015, 21% of the reductions in wheat yield were a consequence of drought on a global scale [6].

Plants have developed multiple adaptive mechanisms to grow under water stress. Some of these mechanisms are stomatal closure, changes in the concentration of plant growth regulators, reduction of vegetative growth, decrease in the aerial/root partitioning ratio, and leaf senescence [7–9]. However, most of these strategies lead to a reduction in biomass accumulation and yield [10]. In wheat, although the yield is defined during the whole cycle, the period from flag-leaf appearance up to 1 week immediately after anthesis is crucial for yield determination and this period is called the “critical period.” During that period, the number of grains, which is the main driver of yield [11, 12], is source-limited, and losses in the capacity to intercept radiation will determine reductions in the number of grains and yield [13].

Water deficit that induces senescence is accompanied by a marked decrease in cytokinin levels [14, 15]. Several researchers have shown that the application of exogenous cytokinin promotes a delay in senescence [16, 17], maintaining the crop capacity for the production of photoassimilates and nitrogen assimilation. Different evidences have also shown that exogenous cytokinin application at different development stages, such as tillering [18], pre-flowering [19], and grain filling [20], modifies grain yield and its components. In this context, the heterologous expression of *isopentenyl transferase* (*IPT*), a gene that encodes the enzyme that catalyzes the rate-limiting step in cytokinin synthesis, increases the endogenous levels of cytokinin and delays leaf senescence in transgenic plants [21–23]. However, when the *IPT* transgene is constitutively expressed, the increased levels of cytokinin induce changes in plant growth, decreasing root development and increasing drought sensitivity [24, 25]. In some species, this issue has been solved by expressing the *IPT* gene under the control of a senescence-associated receptor-like kinase (*SARK*) promoter, which is inducible under maturation [26] or water stress [22]. This strategy curtails the deleterious effects caused by the constitutive expression of *IPT* [27]. In rice, it has been shown that under full irrigation, *pSARK::IPT* transgenic plants did not show differences in biomass, photosynthetic performance, or phenotypic aspects when compared to the controls [27]. However, under water-deficit stress conditions, the expression of *pSARK::IPT* protects rice plants from the adverse effects generated by stress. Similarly, tobacco [22], peanut [28], rice [27], and maize plants [29] exposed to water stress exhibit enhanced photosynthetic capacity, resulting in improved tolerance to water stress. Despite that there was demonstrated a water-stress tolerance of several transgenic crops carrying the expression of *pSARK::IPT* under laboratory and greenhouse conditions [30], very few studies have tested the productivity of transgenic lines in the field [31–34]. Thus, the aims of this work were (i) to obtain *pSARK::IPT* transgenic wheat plants and (ii) to evaluate their behavior under contrasting water availability (drought and well-irrigation) in controlled and field conditions.

## Methods

### Vector construction and plant transformation

**Fig. 1:**
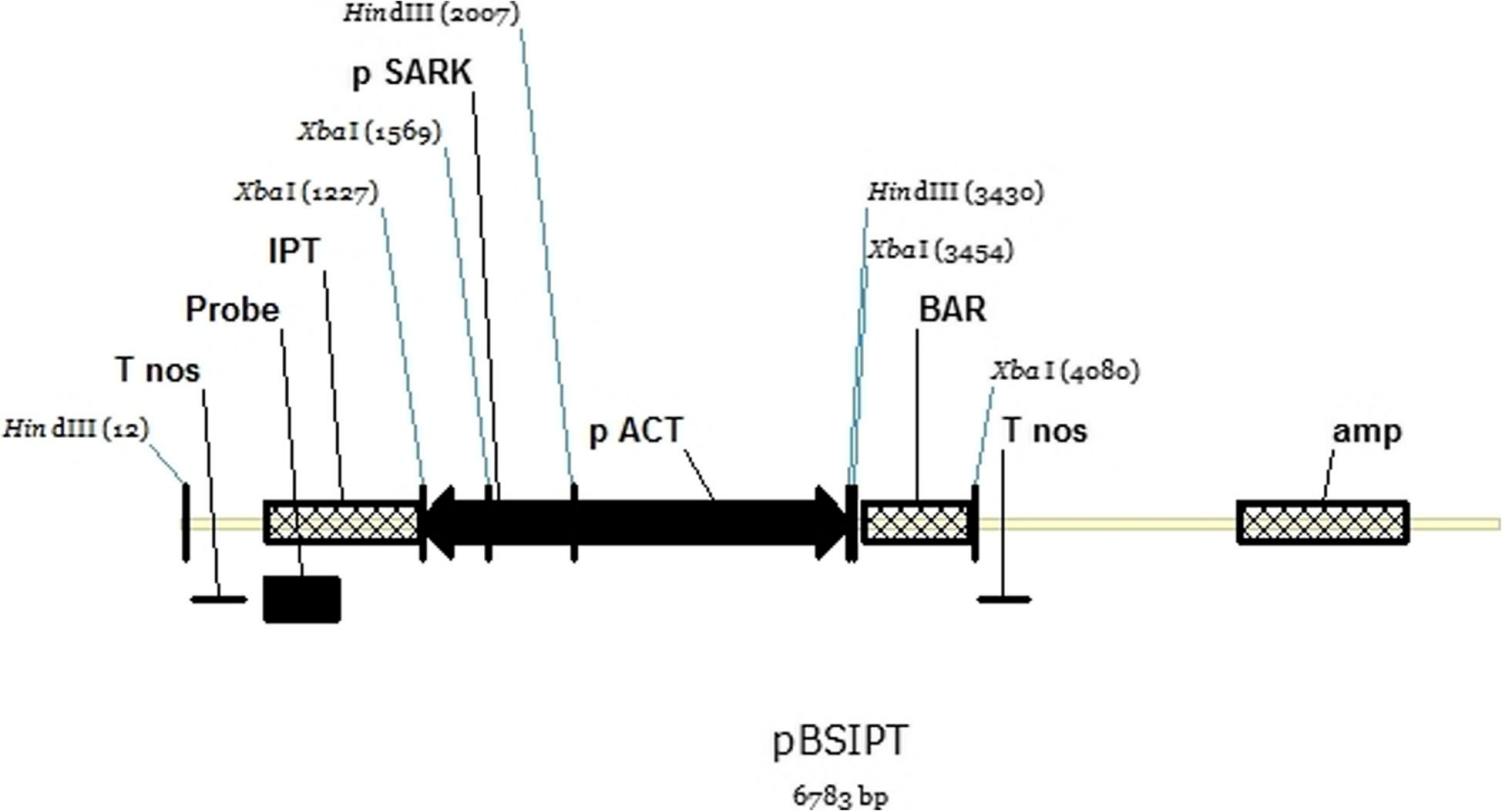
Schematic representation of the plasmid used for the genetic transformation of wheat by biolistics. *IPT*, isopentenyl transferase; p*SARK*, senescence-associated receptor-like kinase promoter; *TNOS*, nopaline synthase terminator; *BAR*, phosphinothricin acetyltransferase; *ACT1*, rice actin promoter

A pBSIPT vector containing the transcription unit *pSARK::IPT* [22], carrying the *IPT* gene from *Agrobacterium tumefaciens* under the regulation of the *SARK* promoter from bean [26], the nopaline synthase terminator [22], and the transcription unit *pACT::BAR*, carrying the phosphinothricin acetyltransferase (*BAR*) gene under the control of the *ACT1* (rice actin) promoter and the nopaline synthase terminator [35], was used for wheat transformation (Fig. 1). *pSARK::IPT* was amplified from a *pSARK::IPT* plasmid [29] by PCR with the oligonucleotides SarkFH (5′ggatctaagcttcttccttagatgctg3′) and IPTRH (5′tttcaaagcttatatatcctgtcaaacac3′), which contain HindIII restriction sites at their ends. Then, this PCR product was introduced as a HindIII fragment into the vector pDM302 [35].

Wheat (*Triticum aestivum* L.) plants (*cv.* ProINTA Federal) were grown in a growth chamber under a 16/8-h photoperiod and 18/15°C day/night air temperature with a light intensity of 190 μmol m^−2^ s^−1^. Scutella were dissected from immature embryos and used for gene transformation, following a biolistic procedure, as described in Pellegrineschi et al. [36].

### Molecular characterization and preliminary stress treatment of transgenic plants

Total genomic DNA was extracted from −70°C frozen wheat leaves using the Dellaporta method [37] for PCR assays and the cetyltrimethylammonium bromide (CTAB) [38] method for Southern blot assays.

The T_0_ transgenic plantlets were tested for the presence of the *IPT* and *BAR* genes by PCR, using the oligonucleotides Sark2201 (5′aagtcaaggtcattggcttaggg3′) and IPT2844 (5′ctcttggtcgggtaacttgtggc3′), and barFw (5′ggatctaccatgagcccaga3′) and barRv (5′tgcctccagggacttcag3′), respectively. All PCR-positive plantlets for both the *IPT* and *BAR* genes were self-pollinated to produce the T1 generation. Flowering time and ex vitro adaptation were recorded.

Plants from all germinated seeds in each plant generation for each experiment were analyzed at the 12 stage (second visible leaf) according to the BBCH code [39] by PCR to identify transgenic individuals from the segregating nulls. Only the selected seedlings were transferred for evaluation in each assay and analysis. As a control, we used WT plants because we did not have enough null segregates to include in our experimental designs (Table S1).

For Southern blot analysis, 20 μg of plant genomic DNA was digested with XbaI (Promega, USA), resolved by electrophoresis in 1% agarose gel, and transferred onto a nylon membrane Hybond N^+^ (Roche, Germany) [39]. The membrane was pre-hybridized for 3 h in hybridization buffer containing 0.2% sodium dodecyl sulfate (SDS), 0.1% sodium N-lauroylsarcosinate, 5X SSC solution (0.75M NaCl, 0.075M sodium citrate), 1% blocker (Roche, Germany), and denatured herring sperm DNA (Promega, USA). A 354-bp probe labeled with digoxigenin-11-deoxyuridine-5′triphosphate (Dig-dUTP; Roche, Germany) was prepared by PCR, using ipt354-Fw (5′caacaagttacccgaccaag3′) and ipt354-Rv (5′tacattccgaacggatgacc3′) oligonucleotides (Fig. 1). The membrane was hybridized overnight at 55°C. The hybridized membrane was washed three times with 2X, 0.2X, and 0.1X of SSC (pH 7.2), respectively, and 0.1% m/v of SDS at 65°C. The chemiluminescent signals were detected on Amersham Hyperfilm^TM^ ECL (Ge, EEUU) and the UVP SYNGENE® system.

For RT-PCR and qRT-PCR, total RNA was extracted from leaf tissues with Trizol® (Invitrogen, USA). The RNA was treated with RQ1 RNase-free DNase (Promega, USA). First-strand complementary DNA (cDNA) was synthesized using oligo(dT)18 and SuperScriptIII Reverse Transcriptase (Invitrogen, USA) with 2 μg of RNA and was performed according to the manufacturer’s instructions.

For RT-PCR, cDNA was amplified by PCR, using oligonucleotides for the *GHS*2 (TgsFw/TgsRv), *BAR* (barFw/barRv), and *IPT* (IptFw/IptRv) genes. The amplification products were analyzed by electrophoresis in 1.5% agarose gels.

An Icycler IQ Real-Time Detection System (BioRad, USA) was used for the qRT-PCR. *TaRP15* is an RNA polymerase 15-kDa subunit gene and was used as a reference gene [40]. The *TaRP15* transcript of 70 bp was amplified with the oligonucleotides TaRPF (5′ gcacacgtgctttgcagataag3′) and TaRPR (5′ gccctcaagctcaaccataact3′). The *IPT* transcript of 70 bp was amplified with the oligonucleotides IPT70-Fw (5′gcagcttgacgcaaatatgg3′) and IPT70-Rv (5′gcgcgcatggatgaaatact3′). Amplification was carried out using the IQ SuperMix-PCR kit (BioRad, USA). The PCR cycling conditions were 1 cycle at 95°C for 5 min, followed by 45 cycles at 95°C for 20 s and 60°C for 40 s. A melting curve was generated by the equipment to assure the specificity of the amplification reaction. For each sample, the reaction was carried out in three replicates. The results were analyzed with a relative standard curve, as described by Bustin [41].

### Drought stress treatments and experimental designs

#### Phenotypic analysis of T1 transgenic wheat plants

For the preliminary phenotypic analysis, a total of 340 seeds from the T1 progeny of six transgenic events (TR1–TR6) and the wild type (WT) were individually planted into 1.2-L plastic pots filled with silty clay loam soil in a growth chamber (Percival Growth Chamber PR1010) [Percival, USA] under a 14/10-h photoperiod and 20/10°C day/night photoperiod with a light intensity of 190 μmol m^−2^ s^−1^. The number of plants for each genotype is described in Table S1. From stage 23 (tillering) to stage 69 (end of flowering), two water treatments were applied: well-watered (WW) and severe water deficit (WD; withholding of watering for 45 days). After the treatment, the pots were well irrigated until maturity. The number of emerged leaves on the main stem, number of tillers, and time to anthesis were recorded.

#### Performance of T2 transgenic wheat plants grown in growth chambers

In experiment 1 (EXP1), seeds from the T2 progeny of the six transgenic events (TR1–TR6) and the WT were imbibed in filter-paper-lined Petri dishes to ensure uniform germination. The selected seedlings by PCR were transferred to 1.5-L pots filled with a mixture of sand and clay loam soil in a ratio of 2:3, fertilized with a 50% N:P:K (20:10:20) solution, and grown in a growth chamber (Percival Growth Chamber PR1010) [Percival, USA]. Temperature and light/dark ratio were increased from 12 to 26°C and from 10/14 (h) to 14/10 (h), respectively, from sowing to ripening to simulate field conditions (Fig. S1), with a light intensity of 190 μmol m^−2^ s^−1^. The different phenological growth stages were recorded using the BBCH code [42]. For the WD treatments, plants were well-watered up to stage 32 (jointing) and then were maintained to a constant 25% field water capacity (FC) from that stage until stage 65 (flowering). Meanwhile, the WW treatment plants were maintained at 70% FC. The experiment was arranged in a factorial combination of genotype × water treatments by using a split-plot design with four replicates (*n*=4). The percentage of FC was controlled by means of a gravimetric soil water content method (Fig. S2a).

For qRT-PCR analyses, leaf tissue samples were taken at stage 65 (flowering) in both WW and WD plants. Time to anthesis was recorded when 50% of the plants in a subplot reached that stage. At maturity, the total above-ground biomass of each pot was individually harvested, and height (from the ground to the top of the ear) was measured. Then, the material was oven-dried at 65°C until constant weight and dry weight were measured. Grain yield, grain number, average individual grain weight, above-ground biomass, and harvest index were determined. The harvest index was calculated as the ratio between grain yield and total above-ground biomass.

#### Performance of T3 transgenic wheat plants in pots growing under greenhouse conditions

Based on their performance in the growth chambers, the transgenic events TR1 and TR4 were selected for two additional experiments (experiments 2 and 3) conducted in a biosafety greenhouse under natural lighting conditions with 20–25°C/12–15°C day/night air temperature.

In experiment 2 (EXP2), seeds from TR1, TR4, and WT genotypes were sown directly (one plant per pot) in 12-L pots containing a mixture of sand and clay loam soil in a ratio of 2:3 and fertilized with a 50% N:P:K (20:10:20) solution. The selected seedlings by PCR were transferred to pots for evaluation. The two water regimens described in the “Performance of T2 transgenic wheat plants grown in growth chambers” section were applied. This experiment was arranged in a factorial combination of genotypes × water treatments by using a randomized design with three replicates (*n*=3). The FC in the pots was controlled by means of a gravimetric soil water content method. Plants were watered daily to keep soil FC at 70%. The WD treatment was applied from stage 47 (late-boot) to stage 65 (flowering) according to the BBCH code [42], and the plants were watered to keep the soil at 25% FC, while the WW treatment was maintained at 70% FC. After the WD treatment, the pots were watered up to 70% FC until harvest (Fig. S2a).

At maturity, the total above-ground biomass of each pot was harvested and separated into the main stem and tillers, and, within each category, biomass was divided into vegetative biomass (shoots plus sheaths and leaves) and spikes. The material was oven-dried as described in the “Performance of T2 transgenic wheat plants grown in growth chambers” section. The number of spikes from the main stem and from tillers was counted and threshed. The average individual grain weight of the main stem and tillers was measured in two sub-samples of 100 grains per category and used to calculate the grain number per spike. Grain yield and its components measured in the whole plant and discriminated for main stems or tillers were recorded.

#### Performance of T3 transgenic wheat plants in microplots growing under greenhouse conditions

Experiment 3 (EXP3) was carried out using the same events that in EXP2 (TR1 and TR4) and WT as a control line, in 1-m^3^ (1m × 1m × 1m) containers (microplots) in the greenhouse under the same conditions described in the “Performance of T3 transgenic wheat plants in pots growing under greenhouse conditions” section. These containers were used to reproduce a micro-crop structure. Seeds of one genotype were sown in one container in rows 15 cm apart (6 rows per container) at a density of 285 pl m^−2^ on 29th May of 2013. The experiment was arranged in a factorial combination of genotypes × water treatments by using a completely randomized design with three replicates (*n*=3). The eighteen containers were filled with a mixture of sand and clay loam soil in a ratio of 2:3. Plants were watered daily to keep the soil near 75% FC until 60 days after sowing. The WD treatment was applied from stage 32 (jointing) to stage 65 (flowering), maintaining the soil at 25% FC. After the WD treatment, all the containers were re-watered up to 75% FC until harvest. The water content throughout the crop cycle was determined by a water probe (MPM160 Moisture Probe Meter, ICT International Pty Ltd., Australia) with a frequency of twice a week (Fig. S2b). A calibration curve (Eq. S1) was built to be able to express the measurements in FC.

At anthesis, five spikes from five different plants per container were selected, and the numbers of spikelets and fertile florets per spikelet were counted to calculate the total number of fertile florets per spike. Florets were considered fertile when the style was curved outwards, and well-developed stigmatic branches were spread wide, with either pollen grains present on them (stage 10 in the scale of Waddington [43]) or anthers green/yellow at anthesis. Then, at crop maturity, the number of grains per spikelet was recorded.

At maturity, total above-ground biomass was collected from 0.5 m of each of two central rows (1 m in total, *ca.* 57 plants) and the same procedure as described in the “Performance of T3 transgenic wheat plants in pots growing under greenhouse conditions” section was followed to record grain yield and its components on the main stem and tillers.

#### Performance of T4 transgenic wheat plants under field conditions

Finally, two field experiments (experiments 4 and 5) were conducted during 2014. Experiment 4 (EXP4) was carried out in Pocito, San Juan province (31° 39.212′ S, 68° 35.249′ W), Argentina, whereas experiment 5 (EXP5) was carried out in Bordenave, Buenos Aires province (31° 39.212′ S, 68° 35.249′ W), Argentina. For both experiments, plants from the T4 progeny from TR1, TR4, and WT were sown. Plants were grown in the field under WW conditions until the start of the WD treatments. In this case, we applied two WD treatments: one from stage 32 (jointing) to stage 65 (flowering) [i.e., during the critical period, WD_CP_] and the other from stage 69 (end flowering) to stage 79 (end of milk development) [i.e., during the grain filling, WD_GF_]. During both WD treatments, the plants were watered to a constant 25% FC, while the WW treatment was maintained at 75% FC (Fig. S2c). The experimental design was a split-plot design where the main plots corresponded to the water treatments and the sub-plots were assigned to the genotypes. In all cases, experiments were carried out with three replicates per treatment in each site (*n*=3). Each plot consisted of five 2-m-long rows 20 cm apart, at a density of 285 seeds m^2^. Soil water content was measured gravimetrically at 0.2 m and 0.4 m depth. Exp 4 was on an Entisol soil (Torrifluvent) of only 0.85 m depth and reduced water holding capacity (126 mm of plant-available soil water) with a pH 7.2–7.7. In Exp 5, soils were sandy loam, classified according to the US Soil Taxonomy as an Haplustol with a pH 7.1. All plots from both experiments received 200 kg N ha^−1^ (100 kg at sowing and 100 kg at *ca.* flowering stage).

At maturity, grain yield and its primary and physiological components were recorded in all treatments, as described in the “Performance of T3 transgenic wheat plants in microplots growing under greenhouse conditions” section. Meteorological data (air minimum and maximum temperature, rainfall, and relative humidity) were recorded every day throughout the crop cycle in both experiments by an automatic meteorological station located in the same site of the experiments.

### Statistical analysis

Analyses of variance (ANOVA) were carried out for physiological traits and grain yield, applying the InfoStat program [44]. The normality of the data was analyzed by the Kolmogorov–Smirnov test. The differences (*p*<0.05) found after conducting a two-way ANOVA were analyzed by least significant difference (LSD) values (*α*=0.1). In cases where the results did not show a normal distribution, a non-parametric analysis (Kruskal–Wallis) was performed. The degree of association between different variables was determined using linear regression models. Pearson’s correlation analysis was conducted to determine the relationship between yield and its primary components. The number of plant generation and the total number of plants used in each experiment are summarized in Table S2.

## Results

### *pSARK::IPT* transgenic plants and selection of transgenic events

**Fig. 2:**
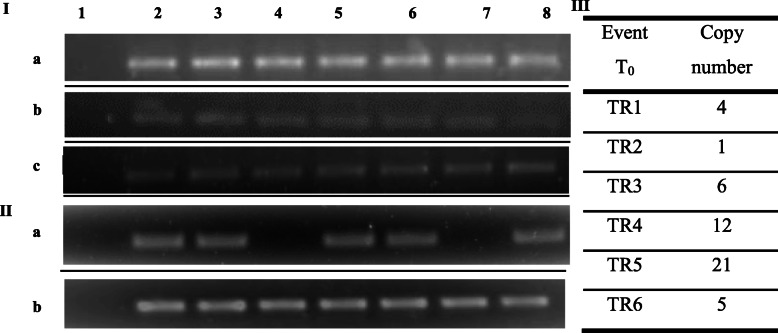
Molecular analysis of transgenic wheat plants. **I** Analysis by PCR of the *IPT* gene (a), *BAR* gene (b), and *GHS* gene (c). Lane 1, negative control; lane 2, positive control; lanes 3–8, six independent putative transgenic plants; all plants had the amplified gene as expected. **II** Analysis by RT-PCR of the *BAR* gene (a) and *GHS* gene (b). Lane 1, negative control; lane 2, positive control; lanes 3–8, six independent putative transgenic plants. **III** Southern blot analysis on T_1_ plants derived from transgenic events, indicating the number of copies of each transgenic event

The pBSIPT vector (Fig. 1) was introduced into the wheat genome (*cv.* ProINTA Federal) by biolistic methods. A total of six independent transgenic plants, called TR1–TR6, were regenerated from 1248 bombarded scutella (transformation efficiency 0.5%). The insertion of the *pSARK::IPT* construct was confirmed by the amplification of the 384-bp and 643-bp PCR fragments of the *IPT* and *BAR* genes, respectively (Fig. 2I (a, b)). Transgene expression of the *BAR* gene was detected in four of the six events (Fig. 2II (a)). Southern blot analysis fully confirmed the PCR results and showed that the number of inserted copies varied across the transgenic plants from one to 21 per genome (Fig. 2III and Fig. S3 (a, b)). All the transgenic events had a similar ex vitro adaptation time (15 days) and flowering time (65 ± 2 days). Furthermore, all of them were fertile, with regular production of self-pollinated T1 seeds.

### Preliminary characterization of T1 plants under severe water stress

**Fig. 3:**
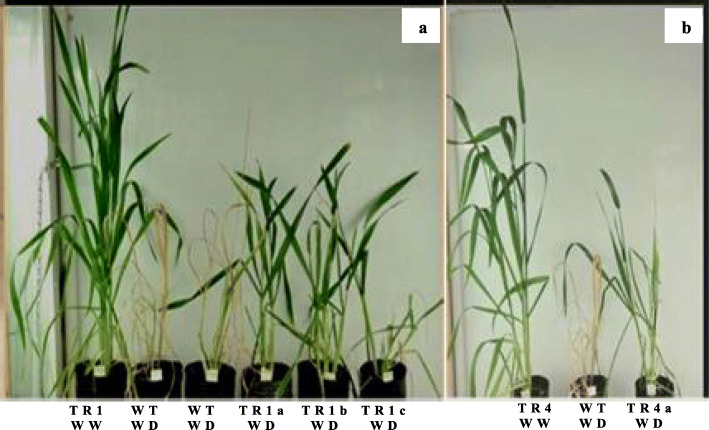
Transgenic wheat plants under WW and WD conditions. **a** Plants from TR1. **b** Plants from TR4. WW: well-watered: WD: water-deficit

For the initial phenotypic characterization, all the plants grown under WD showed a reduction in the number of tillers and the number of emerged leaves. The WT plants did not recover from the severe WD and died. However, plants from events TR1 and TR4 were tolerant and resumed their development after re-watering (Fig. 3).

### Response of the transgenic T2 plants under growth chamber conditions

**Fig. 4:**
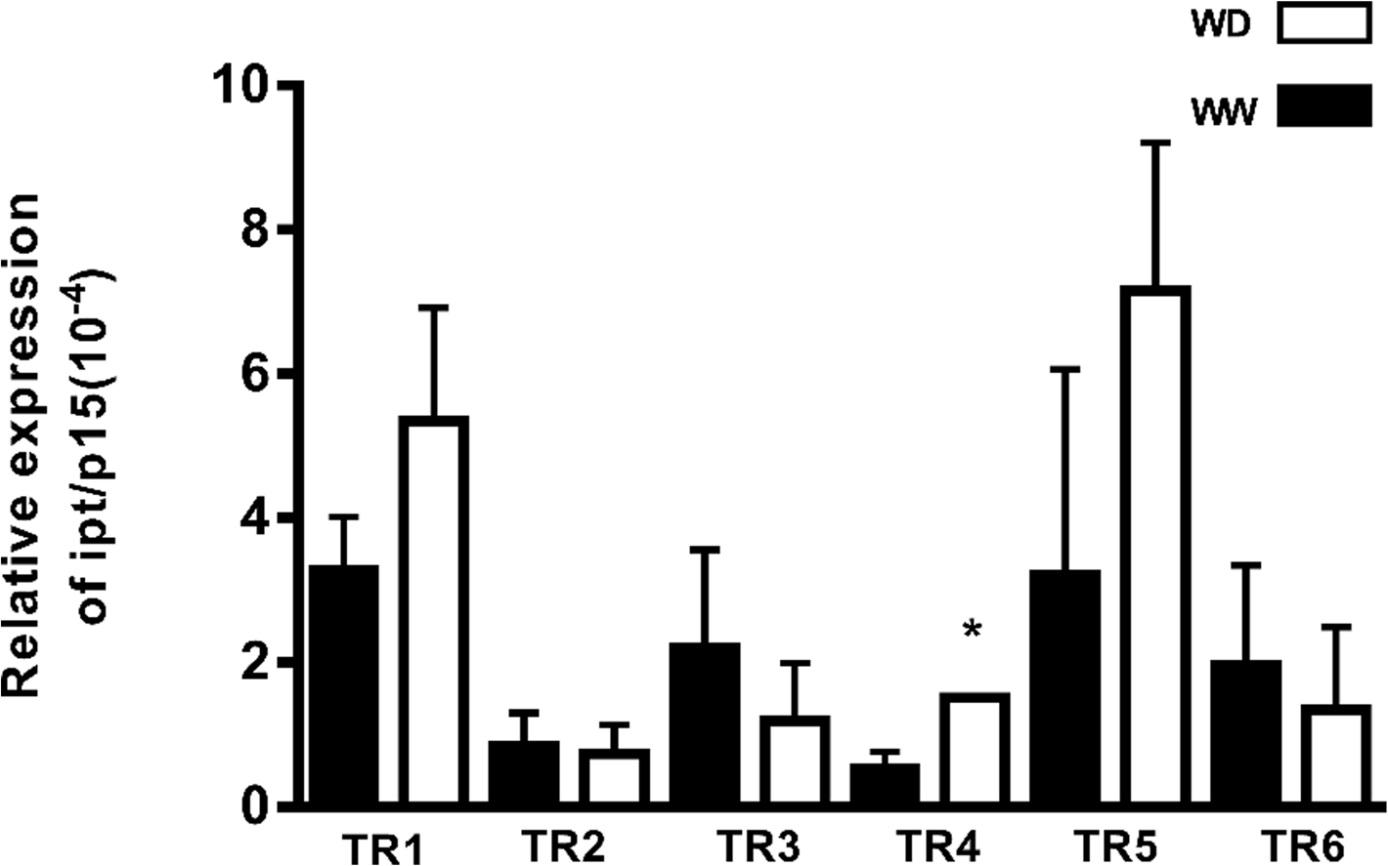
Relative expression of *IPT*/TaRP15 of transgenic events measured by qRT-PCR. The events TR1, TR2, TR3, TR4, TR5, and TR6, measured by qRT-PCR 7 days post-anthesis under well-watered (WW) and water-deficit (WD) conditions. Mean ± S.D., *n* = 4 for each data point. Values expressed at 10^−4^. *p <0.05

*IPT* expression in plants grown under WW and WD conditions at stage 65 (flowering) [at the end of the WD treatment] was compared by qRT-PCR. Although *IPT* expression was higher during WD in TR1, TR4, and TR5, it was statistically different in TR4 (*p*<0.05) (Fig. 4).

The period from seedling emergence to flowering showed significant differences between genotypes (*p*<0.1) and between water treatments (*p*<0.05), with a significant genotype × treatment interaction (*p*<0.005) (Table 1). The flowering time ranged from 63 to 71 days from seedling emergence. WD induced a reduction in the flowering time in all the genotypes evaluated, with a difference of 1–3 days when compared to the WW conditions.

**Table 1:** Agronomic traits of the WT and transgenic events in the growth chamber

Treatment	G	EM-AN (days)	H (cm)	GY (g pl^−1^)	GN	IGW (mg)	B (g)	HI
WW	WT	65^cde^	66.5^b^	6.1^ab^	181.3^a^	34.0^cd^	15.4^bc^	0.40^a^
	TR1	69^ab^	68.0^b^	5.9^ab^	197.3^a^	29.7^cd^	16.2^ab^	0.36^ab^
	TR2	69^bc^	65.3^b^	2.0^fg^	117.5^b^	17.2^e^	9.9^fg^	0.21^de^
	TR3	63^e^	61.8^bc^	6.1^ab^	190.8^a^	32.5^cd^	15.2^bc^	0.40^a^
	TR4	66^bcd^	66.5^b^	6.5^a^	207.8^a^	32.0^cd^	17.8^a^	0.37^a^
	TR5	71^a^	77.8^a^	2.3^ef^	114.6^b^	20.4^e^	15.8^abc^	0.15^ef^
	TR6	63^de^	67.5^b^	5.3^b^	195.8^a^	27.3^d^	15.5^abc^	0.35^ab^
WD	WT	67^ab^	51.3^d^	4.0^c^	111.8^bc^	35.8^bc^	13.4^cde^	0.30^bc^
	TR1	65^bcd^	52.0^d^	4.2^c^	125.8^b^	33.1^cd^	13.8^bcd^	0.30^bc^
	TR2	67^ab^	56.8^cd^	1.3^gh^	41.3^e^	32.4^cd^	10.5^fg^	0.12^f^
	TR3	62^e^	50.0^d^	3.1^d^	75.8^d^	42.3^ab^	9.3^g^	0.35^ab^
	TR4	64^cde^	49.5^d^	3.0^de^	89.8^cd^	34.8^bc^	11.8^def^	0.25^cd^
	TR5	70^ab^	61.6^bc^	1.1^h^	32.4^e^	34.2^c^	9.4^g^	0.11^f^
	TR6	60^e^	41.0^e^	1.7^fgh^	36.8^e^	45.6^a^	10.9^efg^	0.16^ef^
Genotype (G)		***	***	***	***	**	***	***
Water treatment (T)		**	***	***	***	**	***	***
G × T		*	ns	***	***	**	*	ns
LSD		2.7	8.2	0.7	28.1	6.7	2.4	0.06

*G* genotype, *WW* well-watered, *WD* water-deficit conditions, *T* treatment, *EM-AN* days from emergence to anthesis (days), *H* plant height (cm), *GY (g pl*^*−1*^*)* grain yield, *GN* grain number per plant, *IGW (mg)* individual grain weight, *B*, above-ground biomass (g pl^−1^), *HI* harvest index. Asterisks indicate significant differences (*p< 0.1; **p< 0.05; *p*< 0.001; *ns*, not significant). Means (*n*=4) sharing the same letters in columns do not differ (*p*< 0.1)

Four of the six events (TR1, TR3, TR4, and TR6) exhibited no phenotypic differences (i.e., phenology, plant height, number of tillers) with WT plants, independently of the treatment applied. TR2 and TR5 showed unexpected phenotypes. From stage 47 (late-boot), TR2 showed leaf rolling, independently of the treatment applied (Fig. S4a). TR5 showed the most extended seedling emergence-anthesis period and was the tallest genotype, with significant differences (*p*<0.05) with respect to the WT, and also showed an unusual senescence pattern, developing senescence first in the younger leaves instead of the older ones (Fig. S4b).

Grain yield and its components are described in Table 1. The events and water treatments showed significant differences (*p*<0.005) for the numerical and physiological yield components. With the exception of HI, there was a significant G × T interaction (Table 1). The statistical analysis showed a significant negative correlation between grain weight and grain number (*r* = −0.38; *p*<0.01), and the reduction in the grain number was accompanied by a 5–12% increase in grain weight (Table 1). On the other hand, there was no relationship between grain weight and grain yield per plant.

Under WW conditions, TR1 and TR4 plants increased the grain number per plant by 8% and 14%, respectively, compared to the WT, while TR3 and TR6 showed no grain yield penalties (Table 1). Under WD, the TR1 genotype showed the lowest reduction in grain yield (29%), followed by TR3 (49%) and TR4 (53%). Furthermore, TR1 showed the lowest biomass reduction (15%), followed by TR4 (33%) and TR3 (39%) as compared to WT. The lowest yields observed in TR2 and TR5 were a consequence of very low values of harvest index and biomass in both treatments (Table 1).

### Response of T3 plants grown in pots under greenhouse conditions (EXP2)

Grain yield and its components are described in Table 2. When different genotypes were grown under the WW condition, no penalties were associated with the transgenic events. Although no significant differences were observed among genotypes when the plants were grown under WD, the response of the transgenic genotypes differed from that of the WT. Thus, although grain yield per plant was reduced under WD in the three genotypes (*p*<0.05) with respect to the WW treatment, the highest penalty was observed in the WT, with 71% of grain yield reductions, compared to the WW treatment. In both TR1 and TR4, the grain yield penalty was 48% when compared to the WT (Table 2).

**Table 2:** Grain yield and its primary and physiological components for the genotypes grown under greenhouse conditions

		Pots (EXP2)					Microplots (EXP3)				
Treatment	G	GY (g pl^−1^)	GN	IGW (mg)	B (g pl^−1^)	HI	GY (g m^−2^)	GN	IGW (mg)	B (g m^−2^)	HI
WW	WT	32.2^a^	758.9^a^	42.5^bc^	95.6^a^	0.34^a^	463.4^bc^	8651^d^	53.5^a^	1265^a^	0.37^c^
	TR1	31.3^a^	782.5^a^	40.3^c^	93.8^a^	0.33^a^	557.8^a^	11840^b^	47.2^b^	1334^a^	0.42^b^
	TR4	27.8^a^	707.5^a^	40.3^c^	89.1	0.31^a^	501.6^ab^	11760^b^	43.3^c^	1303^a^	0.39^bc^
WD	WT	9.2^c^	183.3^b^	50.4^a^	40.5^b^	0.22^c^	392.9^c^	9029^cd^	43.1^c^	933^c^	0.42^b^
	TR1	16.0^b^	348.0^b^	46.8^ab^	58.9^b^	0.28^b^	537.9^ab^	13693^a^	39.0^d^	1114^b^	0.48^a^
	TR4	14.3^bc^	282.3^b^	50.6^a^	51.4^b^	0.28^b^	422.9^c^	10448^bc^	40.3^cd^	1026^bc^	0.41^b^
Genotype (G)		ns	ns	ns	ns	ns	**	***	***	ns	**
Water treatment (T)		***	***	***	***	***	**	ns	***	***	**
G × T		ns	ns	ns	ns	ns	ns	*	*	ns	ns
LSD		5.9	185.8	5.3	20.5	0.04	75.8	1518	3.7	143.8	0.03
ΔWW vs WD	WT	23.0	575.5	7.9	55.1	0.12	70.5	1387.2	10.5	331.8	0.05
	TR1	15.4	434.4	10.3	37.7	0.06	93.5	1942.5	8.2	277.7	0.07
	TR4	13.5	425.2	6.5	35.0	0.04	104.6	2026.9	3.0	220.1	0.03
Significance		**	*	ns	ns	ns	ns	ns	**	ns	ns

*WW* well-watered, *WD* water deficit, *G* genotype, *T* water treatment, *GY* grain yield, *GN* grain number, *IGW* individual grain number (mg), *B* above-ground biomass, *HI* harvest index. In pots, GY, GN, and B are expressed per plant, whereas in microplots, they are expressed per unit area (m^2^). Asterisks indicate significant differences (*p< 0.1; **p< 0.05; *p*< 0.001; *ns*, not significant). Means (*n*=3) sharing the same letters in columns do not differ (*p*< 0.1)

The analysis of yield components showed that the grain number per plant was positively and significantly correlated with grain yield per plant (*r*=0.98; *p*<0.001). In TR1 and TR4, the exposure to WD affected grain yield more in tillers (50% reduction) than in the main stem (10% reduction) when compared to the WW treatment. A similar response was observed in the grain number (60% reduction), while in main stems, its reduction was 30% when compared to the WW treatment (Table 3).

**Table 3:** Grain yield and its components discriminated for main stems or tillers under greenhouse conditions

Treatment	G	Pots (EXP2)						Microplots (EXP3)					
		GY (g pl^−1^)		GN		IGW (mg)		GY (g m^−2^)		GN		IGW (mg)	
		Ms	Ts	Ms	Ts	Ms	Ts	Ms	Ts	Ms	Ts	Ms	Ts
WW	WT	3.7^a^	28.5^a^	89.7^a^	669.2^a^	41.0^c^	42.8^b^	431.7^b^	31.8^c^	8029.4^d^	621.5^d^	53.7^a^	49.9^a^
	TR1	3.3^ab^	28.0^a^	85.7^a^	696.9^a^	38.4^c^	40.6^b^	493.7^a^	64.1^b^	10546.1^a^	1294.1^cd^	46.8^b^	49.9^a^
	TR4	2.9^bc^	24.9^a^	71.3^b^	636.2^a^	40.5^c^	40.3^b^	423.6^bc^	78.0^ab^	9185.0^c^	2575.3^a^	46.4^bc^	38.0^b^
WD	WT	0.8^d^	8.3^b^	14.3^d^	169.0^b^	57.9^a^	49.8^a^	374.5^cd^	18.4^c^	8448.0^cd^	581.5^d^	43.9^c^	29.7^c^
	TR1	3.0^bc^	13.0^b^	57.0^c^	291.1^b^	53.1^ab^	46.1^ab^	471.9^ab^	66.1^b^	11961.8^b^	1731.1^bc^	39.2^d^	37.2^b^
	TR4	2.8^c^	11.5^b^	53.3^c^	229.1^b^	52.0^b^	50.3^a^	330.9^d^	92.0^a^	8080.4^d^	2368.1^ab^	41.0^d^	37.6^b^
Genotype (G)		**	ns	**	ns	ns	ns	**	**	**	**	*	ns
Water treatment (T)		***	***	***	***	***	***	*	ns	ns	ns	**	**
G × T		***	ns	***	ns	ns	ns	ns	ns	ns	ns	ns	ns
LSD		0.5	5.9	9.8	181.8	5.7	6.32	49.6	21.0	917.8	756.1	2.7	5.3
ΔWW vs WD	WT	2.6	20.2	75.3	500.2	17.0	7.0	58.9	13.3	1273.9	185.6	9.8	20.1
	TR1	0.3	15.0	28.7	407.2	14.7	5.6	85.0	21.3	1742.5	438.4	7.6	12.7
	TR4	0.2	13.4	18.0	405.8	11.5	10.1	92.7	38.9	1165.5	861.4	5.4	3.2
Significance		***	**	***	**	**	ns	ns	**	ns	**	ns	***

*WW* well-watered, *WD* water deficit, *G* genotype, *T* water treatment, *MS* main stems, *TS* tillers, *GY* grain yield, *GN* grain number, *IGW* individual grain number. In pots, GY and GN are expressed per plant, while in microplots, they are expressed per unit area (m^2^). Asterisks indicate significant differences (*p< 0.1; **p< 0.05; *p*< 0.001; *ns*, not significant). Means (*n*=3) sharing the same letters in columns do not differ (*p*< 0.1)

Considering the primary yield components of grain number, when plants were exposed to WD, the WT was the most sensitive genotype, with a reduction in both the number of spikes and the grain number per spike. However, the number of spikes was reduced *ca.* 40% in the WT and only 10% in both transgenic events when WD was compared to WW. The grain number per spike was slightly more sensitive in the WT (with reductions of 63% when compared to the WW treatment) than in TR1 and TR4 (with reductions of 53%) when plants were exposed to WD, compared to those under WW conditions (Table 4).

**Table 4:** Primary subcomponents of the grain number in EXP 2 and EXP3 under greenhouse conditions

Treatment	G	Pots (EXP2)		Microplot (EXP3)	
		NS	GNS	NSA	GNS
WW	WT	11.7^a^	64.9^a^	464.4^b^	18.7^c^
	TR1	13.3^a^	58.5^ab^	468.9^b^	25.4^b^
	TR4	14.7^a^	48.6^b^	535.6^a^	22.0^bc^
WD	WT	7.3^b^	24.0^c^	362.2^c^	25.0^bc^
	TR1	12.7^a^	28.6^c^	408.9^bc^	34.0^a^
	TR4	12.7^a^	22.5^c^	453.3^b^	22.9^bc^
Genotype (G)		**	*	**	**
Water treatment (T)		*	***	***	**
G × T		ns	ns	ns	ns
LSD		3.4	10.4	60.8	6.3
ΔWW vs. WD	WT	4.3	40.9	102.2	6.3
	TR1	1.6	31.3	60.0	8.6
	TR4	2.2	26.1	80.2	1.6
Significance		**	***	ns	**

*G* genotype, *T* water treatment, *WW* well-watered, *WD* water deficit, *NS* number of spikes per plant, *GNS* grain number per spike, *NSA* number of spikes per unit of area. Number of spikes and grain number per spike (GNS) for the three genotypes (WT, TR1, and TR4) grown in pots (EXP2) and microplots (EXP3) in the greenhouse under well-watered (WW) and water-deficit (WD) treatments. In pots, NS is expressed per plant, whereas in microplots, it is expressed per unit area (m^2^). Asterisks indicate significant differences (*p< 0.1; **p< 0.05; *p*< 0.001; *ns*, not significant). Means (*n*=3) sharing the same letters in columns do not differ (*p*< 0.1)

**Fig. 5:**
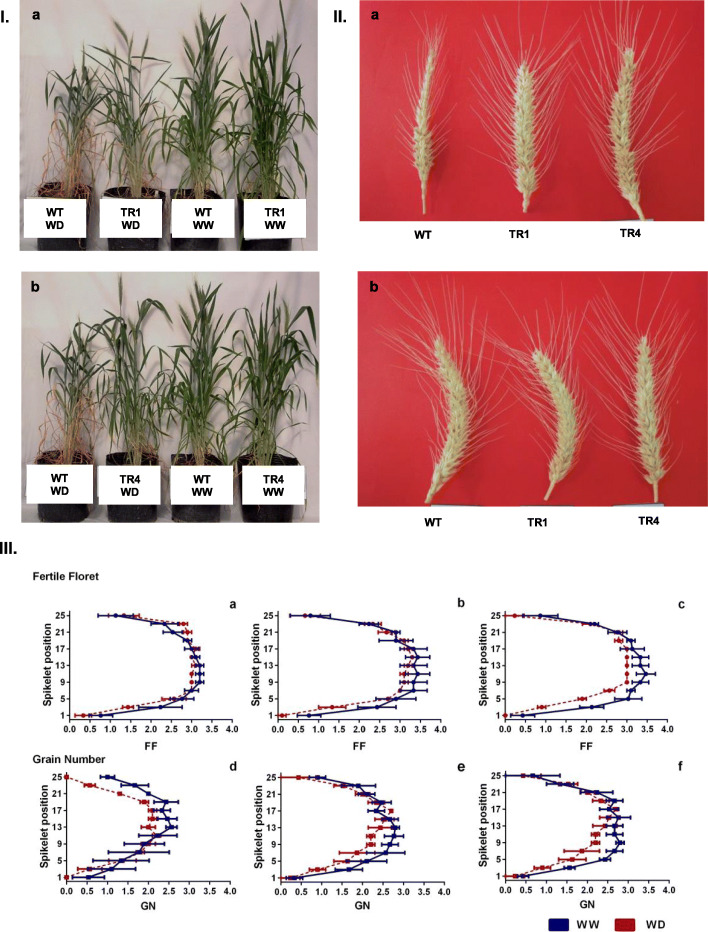
Phenotyping transgenic plants under drought condition in EXP 2 and EXP 3. **I** Plants of the three genotypes under the two water treatments in EXP2. (a) WT vs. TR1 under water deficit (WD) and well-watered (WW) conditions; (b) WT vs. TR4 under WD and WW. **II** Main spike from the genotypes WT, TR1, and TR4 under different treatments in EXP2. (a) WD condition and (b) WW condition. **III** Profile of fertile florets and grain number per spikelet along with the spike (0 corresponds to the basal spikelet) in EXP3 for each genotype (WT, TR1, and TR4) under WD and WW conditions. (a–d) WT; (b–e) TR1; (c–f) TR4. The dotted lines correspond to WD and the line continues to WW conditions. Mean ± S.D., *n* = 15 for each data point

Under WD, the WT showed a significant reduction (*p*<0.05) in grain number per plant (76%), which was significantly correlated (*p*<0.05) with a reduction (63%) in the grain number per spike per plant (Fig. 5II; Table 3). Similarly, the WT also showed a significant reduction in biomass (*p*<0.05) of 58% when exposed to WD, whereas TR1 and TR4 showed reductions of 37% and 42% when compared to the WW treatment (Table 2). Harvest index was more affected in the WT than in the events under WD, with reductions of 15% in TR1, 9% in TR4, and 35% in the WT when plants were grown under WD (Table 2).

### Response of T3 plants grown under greenhouse conditions in microplots (EXP3)

When genotypes were grown in containers using a microplot structure, grain yield significantly differed between genotypes (*p*<0.05) and water treatments (*p*<0.05). TR1 plants showed the highest grain yield in both water conditions. Under the WD treatment, TR1 over-yielded the WT by 37% and TR4 by 8% (Table 2). TR1 also showed less penalty in grain yield, with a reduction of 4% when exposed to WD with respect to the WW conditions, followed by TR4 and WT (Table 2).

The analysis of the grain yield components showed that grain number m^−2^ was the component that best explained the variations in yield (*r* =0.79 in WW *p*<0.05, *r* = 0.91 in WD *p*<0.05) as there was no significant correlation between grain weight and grain yield (*p*>0.1). TR1 and TR4 showed a higher grain number m^−2^ than the WT in both treatments (Table 2). Grain weight was significantly (*p*<0.05) affected by the genotypes, with reductions of around 20% in WT and TR1 and only 7% in TR4 when plants were exposed to WD (Table 2). Biomass showed no significant differences among genotypes, but WD reduced aerial biomass between 18 and 27%. The harvest index increased between 5 and 14% under WD with respect to the WW treatment, with significant differences between genotypes, with TR1 showing the highest values (Table 2).

When the effects of genotype and treatment on grain yield and its components were analyzed in main stem and tillers, the ANOVA showed significant genotype effects on all yield components in both, but different water conditions only significantly affected grain yield in the main stem and grain weight. There was no genotype per treatment interaction for the variables analyzed. TR1 showed the highest grain yield in the main stem, while TR4 showed the highest yield in tillers. When WD was applied, the main stem was reduced between 5% (TR1) and 22% (TR4). However, the most critical differences between genotypes were observed in tillers, while in the WT, grain yield was reduced by 43%; TR1 and TR4 showed no negative impact on tillers when plants were exposed to WD (Table 3). Grain number per unit of area was not affected by WD and followed a trend similar to that observed in grain yield when the main stem was considered in terms of differences between genotypes. WD had a significant effect (*p*<0.005) on grain weight in both main stem and tillers, and the magnitude was dependent on the genotype. In the main stem, TR4 showed the lowest reduction (11%), followed by WT (19%) and TR2 (22%). However, in tillers, the WT showed a dramatic reduction in grain weight (40%), followed by TR1 (19%) and TR4 (2%) (Table 3).

Regarding the fertile florets and grain number per spikelet, the events did not show significant changes in the fertile florets and in the grain number in any spikelet position with respect to the WW conditions (Fig. 5III (b–e, c–f)). Meanwhile, the WT decreased its grain number per spikelet ca. 33% in the middle spikelet positions and a decrease of 100% for the basal and apical positions in the spike. This behavior was consistent with that obtained in EXP2 (Fig. 5II (a, b)).

#### Primary components of the grain number under greenhouse conditions

Under WD, the grain number per spike in pots (EXP2) and microplots (EXP3) showed significant differences (*p*<0.05) (Table 3). When both components of the grain number were analyzed, significant differences were found in genotype and treatment, without a genotype × treatment interaction. In plants grown under WW conditions, the highest grain number was observed in TR4 and the lowest in the WT. A similar genotypic trend was observed in the WD treatments, but the highest reduction (when compared to the WW treatment) was observed in the WT (22%), with respect to the transgenic events (14%; Table 4). The grain number per spike showed the lowest values in the WT when plants were grown under WW conditions, but, under WD, except for TR4, the grain number per spike increased *ca*. 30%, probably due to a compensation effect with a reduction in the number of spikes (Table 4). Changes in the grain number per unit of the area were explained by changes in the grain number per spike (*r*=0.93 (EXP2) and *r*=0.79 (EXP3)) more than by variations in the number of spikes (*r*=0.62 (EXP2) and *r*=0.19 (EXP3)).

### Response of T4 plants grown under field conditions (EXP4–EXP5)

**Fig. 6:**
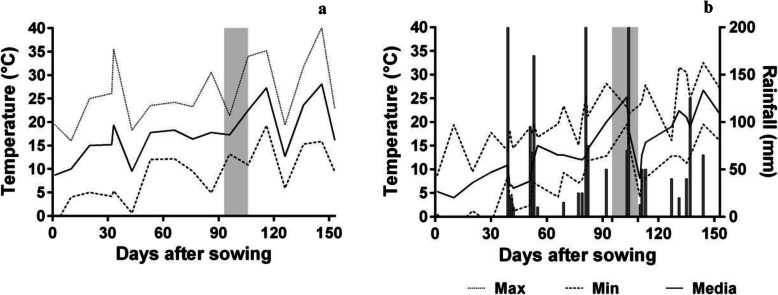
Temperature and rainfall after sowing in the field. **a** San Juan (EXP4). **b** Bordenave (EXP5). The dotted lines correspond to the minimum and maximum temperatures, and the continuous line corresponds to the mean. *The gray zone shows the flowering period

The mean temperature and rainfall for the complete cycle during EXP4 (San Juan) and EXP5 (Bordenave) are shown in Fig. 5. In both experiments, the average air temperature during the preanthesis phase varied from 14 to 25°C. However, the thermal amplitude varied from 6 to 38°C in EXP4 and from 14 to 25°C in EXP5. In EXP5, the rain during the crop cycle was 502 mm, and, as a consequence, the WD_CP_ treatment could not be applied (Fig. 6b).

**Fig. 7:**
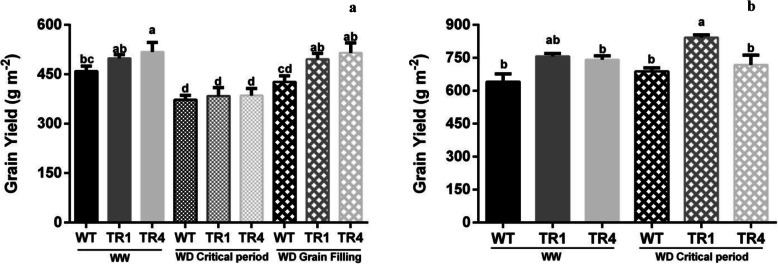
Grain yield for the three genotypes (WT, TR1, and TR4) grown under field conditions. Grain yield obtained in well-watered (WW) and water-deficit conditions applied during the critical period (WD_CP_) and grain filling (WD_GF_) in **a** San Juan (EXP4) and **b** Bordenave (EXP5). Mean ± S.D., *n* = 3 for each data point. Means sharing the same letters in columns do not differ (*p*< 0.1)

Grain yield, grain number, and grain weight showed significant differences among genotypes in both EXP4 and EXP5. However, when both physiological components of yield (i.e., above-ground biomass and harvest index) were considered, significant differences among genotypes were only evident in EXP4 (Table 5, Fig. 7).

**Table 5:** Grain yield and its primary and physiological components under field conditions

Treatment	G	San Juan (EXP4)					Bordenave (EXP5)				
		GY (g m^−2^)	GN	IGW (mg)	B (g m^−2^)	HI	GY (g m^−2^)	GN	IGW (mg)	B (g m^−2^)	HI
WW	WT	455^bc^	15620^b^	29.2^bc^	1056^bc^	0.43^b^	701^b^	18887^b^	37.1^ab^	1516^b^	0.46^b^
	TR1	505^ab^	18521^a^	27.4^cd^	1252^a^	0.40^b^	767^ab^	20938^ab^	36.6^bc^	1602^ab^	0.48^a^
	TR4	518^a^	19646^a^	26.8^d^	1103^bc^	0.48^a^	719^b^	20381^ab^	35.3^cd^	1565^b^	0.46^b^
WD_CP_	WT	373^d^	11070^c^	33.7^a^	883^d^	0.42^b^	695^b^	18145^b^	38.6^a^	1526^b^	0.45^b^
	TR1	384^d^	11811^c^	32.4^a^	899^d^	0.43^b^	844^a^	23060^a^	36.6^bc^	1807^a^	0.47^ab^
	TR4	386^d^	11630^c^	33.1^a^	992^cd^	0.39^b^	710^b^	20686^ab^	34.7^d^	1557^b^	0.46^b^
WD_GF_	WT	425^cd^	14388^b^	29.7^b^	1087^c^	0.40^b^					
	TR1	490^ab^	18116^a^	28.2^d^	1223^ab^	0.42^b^					
	TR4	504^ab^	18154^a^	28.2^cd^	1236^ab^	0.44^b^					
Genotype (G)		**	***	***	ns	ns	**	**	***	*	***
Water treatment (T)		***	***	**	**	ns	ns	ns	ns	ns	ns
G × T		ns	ns	ns	ns	**	ns	ns	ns	ns	ns
LSD		57.5	1951	1.91	133.5	0.03	78.0	2460	1.32	180.4	0.01

*G* genotype, *T* water treatment, *GY* grain yield (g m^−2^), *GN* grain number per unit area, *IGW* individual grain weight (mg), *B* above-ground biomass (g m^−2^), *HI* harvest index. Primary components for the three genotypes (WT, TR1, and TR4) grown under field conditions in well-watered (WW) and water-deficit conditions applied during the critical period (WD_CP_) and grain filling (WD_GF_) in San Juan (EXP4) and Bordenave (EXP5). Asterisks indicate significant differences (*p< 0.1; **p< 0.05; *p*< 0.001; *ns*, not significant). Means (*n*=4) sharing the same letters in columns do not differ (*p*< 0.1)

Both the genotypic differences and the treatment effects on grain yield were significantly related to the grain number (EXP4: *r*=0.88 and EXP5: *r*=0.94, *p*<0.001), but no relationship between grain weight and grain yield was found. Moreover, the statistical analysis showed a significant negative correlation between average individual grain weight and grain number (EXP4: *r*= −0.70, *p*<0.001; EXP5: *r*=−0.48, *p*<0.05).

In these field conditions, transgenic plants with the TR1 and TR4 events showed higher grain yield and grain number than the WT (Table 5). In EXP4, both the WD applied during the critical period (WD_CP_) and during the grain filling (WD_GF_) produced grain yield penalties that were mostly associated with decreases in grain number, especially under WD_CP_, during which grain yield was significantly reduced (*p*<0.001) in all genotypes. Under WD_GF_, the grain number was reduced by 6%. Associated with the grain number reduction, grain weight was significantly increased for all genotypes in WD_CP_, with the highest increases in TR4 (24%), followed by TR1 (18%). In contrast, the average individual grain weight was not significantly modified when crops were grown under both WW and WD_GF_ conditions. In EXP5, grain yield and its primary components were not significantly modified by the treatments due to the excessive precipitation. Based on the grain yield obtained in each experiment, TR1 over-yielding the WT in both environments in each condition.

Regarding the physiological components of grain yield in EXP4, crops exposed to WD_CP_ produced lower biomass during the whole cycle (10–28%; *p*<0.05), while the harvest index was unaffected by both WD treatments when compared to the WW treatment. No significant differences were observed between genotypes, although, in both treatments, TR1 and TR4 showed a trend to produce more biomass (from 10 to 14%) than the WT (Table 5). In EXP5, the biomass and harvest index were significantly affected by the genotype, but there was not a consistent pattern between them; TR1 showed the highest biomass in the WD_CP_ while the WT recorded the lowest values of harvest index.

### Correlation between the primary components of yield under both microplots and field conditions

The correlation coefficients calculated between the primary components of yield for microplots and fields are shown in Table S3. Interestingly, under both WW and WD regimes, grain yield differences between genotypes were explained mainly by the grain number and showed a significant positive correlation with grain yield (WW: *r*=0.78, *p*<0.001; WD: *r*=0.74, *p*<0.001), and no significant relationship was found with grain weight.

When dissecting the grain number into its components, both the grain number per spike and the number of spikes explained the combined effects of treatments on the grain number. Both traits showed the strongest correlation under both WW (grain number per spike *r*=0.87, *p*<0.001; number of spikes per unit of area: *r*=0.82, *p*<0.001) and WD conditions (grain number per spike *r*=0.74; number of spikes per unit of area: *r*=0.75, *p*<0.001). As a consequence of the relationship between grain yield and grain number, a positive correlation was found between grain yield and grain number per spike (*r*=0.56) and grain yield with the number of spikes per unit of area (*r*=0.72) under WW conditions. In contrast, under WD conditions, variations in grain yield were positively correlated only with changes in the grain number per spike (*r*=0.81) and not with the number of spikes. Both of its primary components jointly contributed to the increase in grain yield.

Regarding grain weight, a negative correlation was found with the grain number under both WW (*r*=−0.66, *p*<0.001) and WD conditions (*r*=−0.61, *p*<0.005). Thus, this negative relationship could be explained by a negative correlation with the number of spikes per unit of area (WW: *r*=−0.52, *p*<0.001; WD: *r*=−0.83, *p*<0.001) and the grain number per spike under WW conditions (*r*=−0.75, *p*<0.001).

## Discussion

In cereal crops such as wheat, abiotic stresses as water deficit, high temperatures, and salinity are the main causes of yield losses [9]. Thus, in the last decades, there have been obtained transgenic plant crops able to mitigate the deleterious effects of these abiotic stresses [45, 46]. Pellegrineschi et al. [47], for example, expressed the *DREBs/CBFs* genes, increasing the expression of genes associated with high levels of sugars and proline, increasing the tolerance to water stress. Sivamani et al. expressed the *HVA1* gene in barley, improving water use efficiency, biomass accumulation, and root development [48]. Abebe et al. increased drought tolerance by expressing the *mtlD* gene, a gene encoding enzymes mediating mannitol biosynthesis [49], and Vendruscolo et al. reported that the expression of the gene encoding P5CS, a regulatory enzyme mediating proline biosynthesis, increased the plant tolerance to water deficit, mainly due to the role of proline as an antioxidant [50]. However, despite the emphasis and prospects generated, studies on transgenic events in field-grown plots are scarce and usually fail to demonstrate the expected phenotype [33, 45]. As far as we know, one of the few studies carried out under field conditions was published by Gonzalez et al. who showed that transgenic wheat carrying a mutated version of the *HaHB4* gene had improved drought resistance under field conditions [34].

In the present work, we demonstrated that the expression of the *IPT* gene, driven by the inducible *SARK* promoter, contributed to reducing the penalty in grain yield under water deficit in transgenic wheat plants, not only under controlled conditions but also under field conditions, which is consistent with results obtained in other species transformed with *pSARK::IPT* [22, 25, 28, 51]. Moreover, our results showed that plants transformed with *pSARK::IPT* increased grain yield under well-watered field conditions, as previously reported in transgenic peanut expressing *pSARK::IPT* [28].

Molecular analysis of the six transgenic wheat events here obtained showed differential gene insertion patterns, different patterns of expression, and differences in morphology, physiology, and yield levels. Such varied responses to the insertion of a single gene have been extensively described [52]. Also, molecular, morphological, and physiological changes are produced by minimal modifications in expression levels generated by the insertion of transgenes into key metabolic pathways [53], transcription factors [30, 54], or enzymes that limit the production of hormones [55, 56].

Crop yield is a complex trait that depends on both genetic and environmental parameters [57, 58]. In the present study, the events expressing *IPT* showed a water-deficit-tolerant phenotype in different environments. Transgenic TR1 and TR4 wheat events displayed delayed senescence, similar to the reported effect of *IPT* expression in other species [22, 27–29], and thereby displayed a lower yield penalty under water-deficit stress both in the greenhouse and in the field experiments.

The effect of water stress on reproductive processes in cereals has been exhaustively studied [59, 60]. Water stress during preanthesis causes a dramatic effect on the number of grains [61–63], particularly during the “critical period” (i.e., from middle stem elongation to 1 week after anthesis) because, during this period, the number of grains is established through the two primary components associated with the grain number: grain number per spike and number of spikes [11, 12]. Notably, the main primary yield component that explained the differences in grain yield in the transgenic plants with respect to the WT was the grain number and not the grain weight.

Different evidences have shown that water stress reduces the grain number per spike, which is linked to the development of the reproductive organs [59, 64, 65]. It has also been described that floral abortion is a consequence of a decrease in the water potential and an increase in the accumulation of abscisic acid (ABA) [66], causing a lower capacity of the establishment of fertile flowers and directly affecting the number that will be established as grains. In corn plants expressing *IPT*, Décima Oneto et al. showed a decrease in ABA, suggesting that this behavior could explain the better performance of transgenic plants grown under WD, when compared to the controls, maintaining the seed set as a consequence of reducing the number of aborted flowers and thereby the grain number per spike. In the present work, the advantages in grain number (per plant and unit area) in transgenic plants when compared to the WT were associated with both the grain number per spike and the number of spikes [51]. Increases in the grain number due to a higher number of spikes have also been reported by Koprna et al. after the exogenous application of cytokinin [18].

The increases in the grain number per spike in transgenic plants (when compared to the control) were probably associated with an increased floret fertility survival, promoting a higher number of fertile florets at anthesis, more than changes in the maximum number of total floret primordia [67]. The same reasoning could be applied for the increases in the number of spikes per plant, being significant in the TR4 event regardless of the treatment applied. In this sense, the transgenic genotypes presented a higher number of spikes than the WT genotype but without a complete compensation with the grain number per spike. Thus, the increase in one primary component could determine a partial trade-off in the other, and thereby the final result is a positive effect on the grain number as the counterbalance between components is only partial.

## Conclusion

The expression of the *IPT* gene regulated by the *SARK* promoter in wheat allowed to mitigate the damage caused by water-deficit stress and improving plant productivity under normal watering conditions. Moreover, its expression in wheat did not modify phenological growth stages and did not penalize the grain yield even when plants were grown under well-watered conditions. Nowadays, the increase in grain yield as a result of conventional breeding is estimated at about 0.5%. So, the insertion of the *IPT* gene under the control of the *SARK* promoter could be an interesting tool for breeding and this process may generate a genetic advance in yield. Thus, TR1 and TR4 seem to be promising events under a wide range of water conditions, especially when plants are exposed to water-stress limitation.

## Supplementary Information

**Additional file 1: Supplementary Fig. 1.** Mean daily temperature and photoperiod of Experiment 1 (EXP1) in the growth chamber (Percival Growth Chamber PR1010) during the whole plant’s cycle. DE: Days from emergence.

**Additional file 2: Supplementary Fig. 2.** Percentage of water content in the soil in each experiment: (a) pots in the growth chamber (EXP1) and greenhouse (EXP2); (b) microplots (EXP3); and (c) field in San Juan province (EXP4).

**Additional file 3: Supplementary Fig. 3.** Southern blot analysis on T_1_ self-pollinated plants derived from transgenic events. The genomic DNA was digested with Xba I; each band is considered an insertion site. **(a)** TR1 event: lane 1 wild type (ProINTA Federal) control; lanes 2-4: different plants of the TR1 event. MW: DNA molecular weight marker II, Digoxigenin labeled. **(b)** TR2, TR3, TR4, TR5, and TR6 events. Lanes 1-2, TR2; lanes 3-4, TR3; lanes 5-6, TR4; lanes 7-8, TR5; lanes 9-10, TR6. MW: DNA Molecular Weight Marker III, Digoxigenin labeled. All plants of the same event have the same gene insertion pattern, as expected.

**Additional file 4: Supplementary Fig. 4.** Transgenic wheat plants in Exp 2. (**a**) Plants from TR2. (**b**) Plants from TR5.

**Additional file 5: Supplementary Table. 1.** PCR analysis of T1 transgenic plants for IPT gene.

**Additional file 6: Supplementary Table. 2.** Trial acronyms, Generation and Total of plants used.

**Additional file 7: Supplementary Table. 3.** Pearson’s correlation coefficients between the primary components of yield in microplots and in the field. WW: well-watered; WD: water deficit; GY: grain yield; GN: grain number; IGW: individual grain weight; GNS: grain number per spike; NSA: number of spikes per unit of area. ns: not significant; * *p* 0.05; ** 0.005; ***: 0.0001.

**Additional file 8: Supplementary Equation 1.** Calibration curve to express the measurements in Field capacity.

## Data Availability

The datasets supporting the conclusions of this article are included within the article and its additional files.

## Abbreviations

IPT: Isopentenyl transferase
SARK: Senescence-associated receptor-like kinase
BAR: Phosphinothricin acetyltransferase
EXP: Experiment
FC: Field water capacity
WW: Well-watered
WD: Water deficit
WD_CP_: Water deficit during the critical period
WD_GF_: Water deficit during grain filling
