# Supplementary material for: Stress-induced expression of IPT gene in transgenic wheat reduces grain yield penalty under drought
Source: J Genet Eng Biotechnol. 2021 May 10;19:67. doi: 10.1186/s43141-021-00171-w (PMC8110665; doi:10.1186/s43141-021-00171-w)

Supplementary Fig. 1. Mean daily temperature and photoperiod of Experiment 1 (EXP1) in the growth chamber (Percival Growth Chamber PR1010) during the whole plant's cycle. DE: Days from emergence.


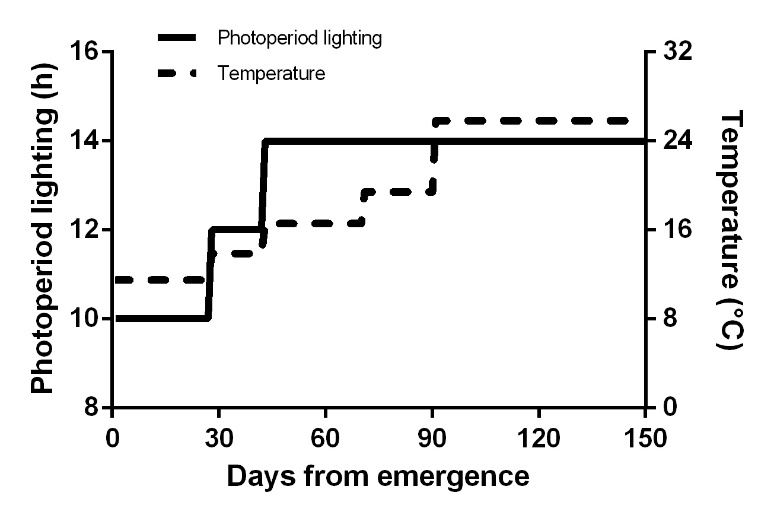

Supplement: Supplementary file 1 — Additional file 1: Supplementary Fig. 1. Mean daily temperature and photoperiod of Experiment 1 (EXP1) in the growth chamber (Percival Growth Chamber PR1010) during the whole plant’s cycle. DE: Days from emergence. [file 43141_2021_171_MOESM1_ESM.docx]
