# Supplementary material for: Stress-induced expression of IPT gene in transgenic wheat reduces grain yield penalty under drought
Source: J Genet Eng Biotechnol. 2021 May 10;19:67. doi: 10.1186/s43141-021-00171-w (PMC8110665; doi:10.1186/s43141-021-00171-w)
Supplement: Supplementary file 2 — Additional file 2: Supplementary Fig. 2. Percentage of water content in the soil in each experiment: (a) pots in the growth chamber (EXP1) and greenhouse (EXP2); (b) microplots (EXP3); and (c) field in San Juan province (EXP4). [file 43141_2021_171_MOESM2_ESM.docx]

Supplementary Fig. 2. Percentage of water content in the soil in each experiment: (a) pots in the growth chamber (EXP1) and greenhouse (EXP2); (b) microplots (EXP3); and (c) field in San Juan province (EXP4).

WD: Water deficit; WW: well water
