# Supplementary material for: Stress-induced expression of IPT gene in transgenic wheat reduces grain yield penalty under drought
Source: J Genet Eng Biotechnol. 2021 May 10;19:67. doi: 10.1186/s43141-021-00171-w (PMC8110665; doi:10.1186/s43141-021-00171-w)
Supplement: Supplementary file 3 — Additional file 3: Supplementary Fig. 3. Southern blot analysis on T1 self-pollinated plants derived from transgenic events. The genomic DNA was digested with Xba I; each band is considered an insertion site. (a) TR1 event: lane 1 wild type (ProINTA Federal) control; lanes 2-4: different plants of the TR1 event. MW: DNA molecular weight marker II, Digoxigenin labeled. (b) TR2, TR3, TR4, TR5, and TR6 events. Lanes 1-2, TR2; lanes 3-4, TR3; lanes 5-6, TR4; lanes 7-8, TR5; lanes 9-10, TR6. MW: DNA Molecular Weight Marker III, Digoxigenin labeled. All plants of the same event have the same gene insertion pattern, as expected. [file 43141_2021_171_MOESM3_ESM.docx]

**Supplementary Fig. 3.** Southern blot analysis on T_1_ self-pollinated plants derived from transgenic events. The genomic DNA was digested with Xba I; each band is considered an insertion site. **(a)** TR1 event: lane 1 wild type (ProINTA Federal) control; lanes 2-4: different plants of the TR1event. MW: DNA molecular weight marker II, Digoxigenin labeled. **(b)** TR2, TR3, TR4, TR5, and TR6 events. lanes 1-2, TR2; lanes 3-4, TR3; lanes 5-6, TR4; lanes 7-8, TR5; lanes 9-10, TR6. MW: DNA Molecular Weight Marker III, Digoxigenin labeled..

**23100**

**9400**

**6600**

**4400**

**2400**

**2100**


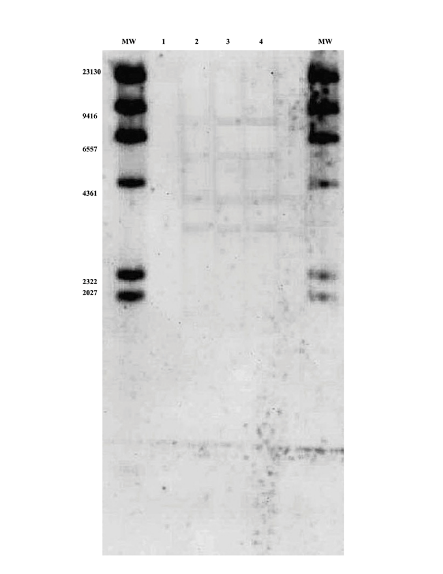


**a**

**21200**

**5100**

**4900**

**4200**

**3500**

**2000**

**1900**

**1600**

**1400**

**1000**

**800**

**500**

**100**


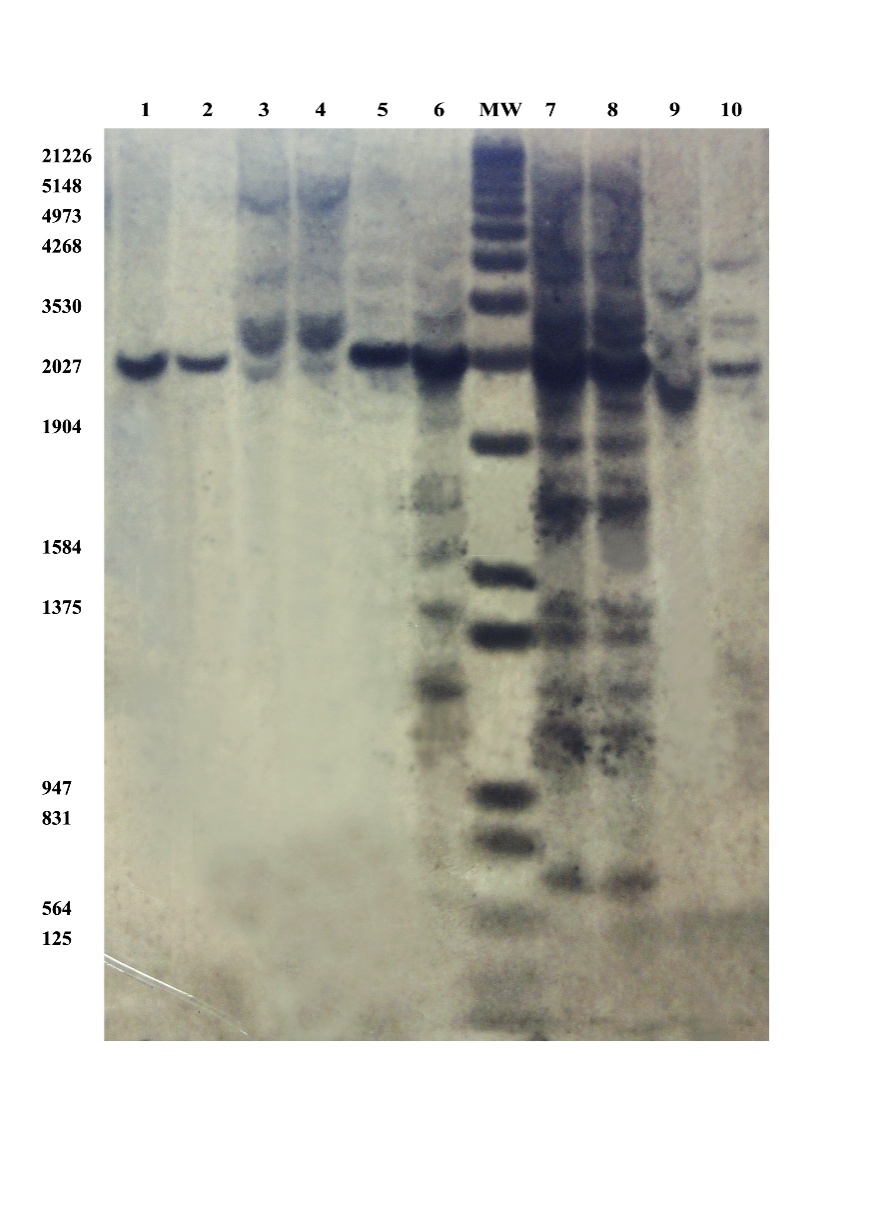


**b**
