# Supplementary figures and images for: Stress-induced expression of IPT gene in transgenic wheat reduces grain yield penalty under drought
Source: J Genet Eng Biotechnol. 2021 May 10;19:67. doi: 10.1186/s43141-021-00171-w (PMC8110665; doi:10.1186/s43141-021-00171-w)

**Supplementary Fig. 4.** Transgenic wheat plants in Exp 2. (**a**)Plants from TR2. (**b**) Plants from TR5


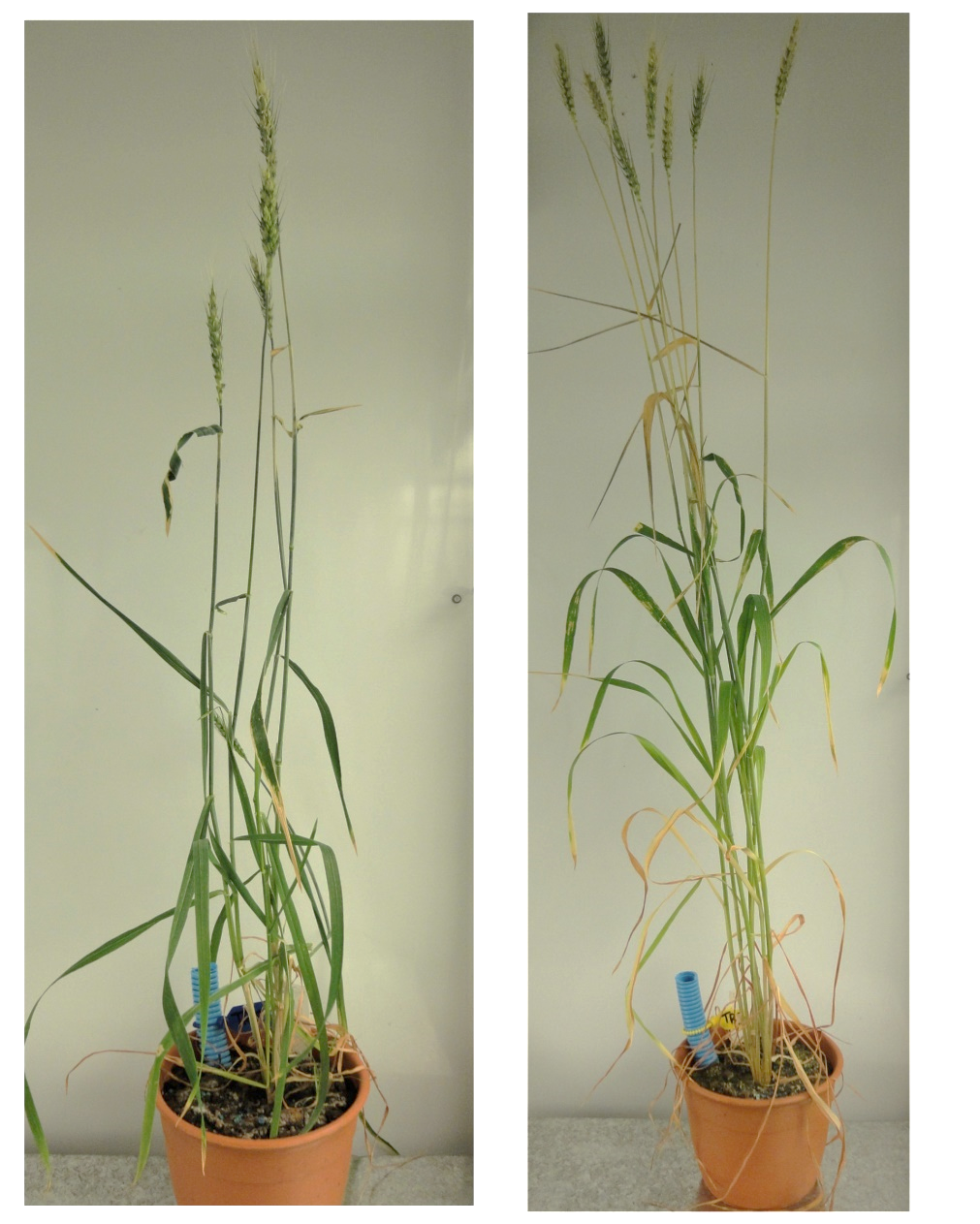


**a**

**b**

Supplement: Supplementary file 4 — Additional file 4: Supplementary Fig. 4. Transgenic wheat plants in Exp 2. (a) Plants from TR2. (b) Plants from TR5. [file 43141_2021_171_MOESM4_ESM.docx]
