# Supplementary material for: Stress-induced expression of IPT gene in transgenic wheat reduces grain yield penalty under drought
Source: J Genet Eng Biotechnol. 2021 May 10;19:67. doi: 10.1186/s43141-021-00171-w (PMC8110665; doi:10.1186/s43141-021-00171-w)
Supplement: Supplementary file 5 — Additional file 5: Supplementary Table. 1. PCR analysis of T1 transgenic plants for IPT gene. [file 43141_2021_171_MOESM5_ESM.docx]

**Supplementary Table. 1.** PCR analysis of T1 transgenic plants for IPT gene.

| **Event** | **Number of T1 seedlings** | | |
| --- | --- | --- | --- |
|  | **Total** | **IPT ^+^** | **IPT^-^** |
| **TR1** | 65 | 60 | 5 |
| **TR2** | 16 | 12 | 4 |
| **TR3** | 66 | 59 | 7 |
| **TR4** | 57 | 52 | 5 |
| **TR5** | 15 | 15 | 0 |
| **TR6** | 67 | 56 | 11 |
