# Supplementary material for: Stress-induced expression of IPT gene in transgenic wheat reduces grain yield penalty under drought
Source: J Genet Eng Biotechnol. 2021 May 10;19:67. doi: 10.1186/s43141-021-00171-w (PMC8110665; doi:10.1186/s43141-021-00171-w)
Supplement: Supplementary file 6 — Additional file 6: Supplementary Table. 2. Trial acronyms, Generation and Total of plants used. [file 43141_2021_171_MOESM6_ESM.docx]

**Supplementary Table. 2.** Trial acronyms, Generation and Total of plants used.

| **Trial** | **Trial Code** | **Generation** | **Total of plants** |
| --- | --- | --- | --- |
| **Preliminary characterization** | - | T1 | 340 |
| **Pots in Growth Chamber** | EXP1 | T2 | 56 |
| **Pots in glasshouse** | EXP2 | T3 | 18 |
| **Microplot in glasshouse** | EXP3 | T3 | 5130 |
| **Field San Juan** | EXP4 | T4 | 15390 |
| **Field Bordenave** | EXP5 | T4 | 10260 |
