# Supplementary material for: Stress-induced expression of IPT gene in transgenic wheat reduces grain yield penalty under drought
Source: J Genet Eng Biotechnol. 2021 May 10;19:67. doi: 10.1186/s43141-021-00171-w (PMC8110665; doi:10.1186/s43141-021-00171-w)
Supplement: Supplementary file 7 — Additional file 7: Supplementary Table. 3. Pearson’s correlation coefficients between the primary components of yield in microplots and in the field. WW: well-watered; WD: water deficit; GY: grain yield; GN: grain number; IGW: individual grain weight; GNS: grain number per spike; NSA: number of spikes per unit of area. ns: not significant; * p 0.05; ** 0.005; ***: 0.0001. [file 43141_2021_171_MOESM7_ESM.docx]

**Supplementary Table. 3.** Pearson’s correlation coefficients between the primary components of yield in microplots and in the field.

|  |  |  | WW | | | | |
| --- | --- | --- | --- | --- | --- | --- | --- |
|  |  | | GY | GN | IGW | GNS | NSA |
| WD | GY | |  | 0.78 *** | -0.08 ns | 0.56 ** | 0.72 *** |
|  | GN | | 0.74 *** |  | -0.66 *** | 0.87 *** | 0.82 *** |
|  | IGW | | 0.07 ns | -0.61 ** |  | -0.75 *** | -0.52 ** |
|  | GNS | | 0.81 *** | 0.74 *** | -0.19 ns |  | 0.76 *** |
|  | NSA | | 0.24 ns | 0.75*** | -0.83 ** | 0.24 ns |  |

WW: well-watered; WD: water deficit; GY: grain yield; GN: grain number; IGW: individual grain weight; GNS: grain number per spike; NSA: number of spikes per unit of area. ns: not significant; * p 0.05; ** 0.005; ***: 0.0001
