# Supplementary material for: Stress-induced expression of IPT gene in transgenic wheat reduces grain yield penalty under drought
Source: J Genet Eng Biotechnol. 2021 May 10;19:67. doi: 10.1186/s43141-021-00171-w (PMC8110665; doi:10.1186/s43141-021-00171-w)
Supplement: Supplementary file 8 — Additional file 8: Supplementary Equation 1. Calibration curve to express the measurements in Field capacity. [file 43141_2021_171_MOESM8_ESM.docx]

**Supplementary Equation1**

Calibration curve to express the measurements in Field capacity.

The values recorded were used to calculate FC % as follows:

FC= [(W_w_-W_dry_) * 100%] / ( X * F_corr_)

where W_w_ is the current wet weight; W_dry_ is the dry weight of the soil mixture before being saturated; X is the g of water uptaken per 100 g of mixture; F_corr_ is a correction factor expressed in g^-1^ to normalize all pots.
